# Post-surgical fall risk prediction: a machine learning approach for spine and lower extremity procedures

**DOI:** 10.3389/fmed.2025.1574305

**Published:** 2025-04-15

**Authors:** Ya-Huei Chen, Xing-Yu Luo, Chia-Hui Chang, Chen-Tsung Kuo, Sou-Jen Shih, Mei-Yu Chang, Mei-Rong Weng, I-Chieh Chen, Ying-Lin Hsu, Jia-Lang Xu

**Affiliations:** ^1^Department of Nursing, Taichung Veterans General Hospital, Taichung, Taiwan; ^2^Department of Institute of Statistics, National Chung Hsing University, Taichung, Taiwan; ^3^College of Nursing, Hungkuang University, Taichung, Taiwan; ^4^Department of Information Management, Taipei Veterans General Hospital, Taipei, Taiwan; ^5^Department of Nursing, Dajia Li Hospital, Taichung, Taiwan; ^6^Department of Research, Taichung Veterans General Hospital, Taichung, Taiwan; ^7^Department of Applied Mathematics, National Chung Hsing University, Taichung, Taiwan; ^8^Department of Computer Science and Information Engineering, Chaoyang University of Technology, Taichung, Taiwan

**Keywords:** falls, classification problems, logistic regression, random forests, health care

## Abstract

In Taiwan, two key indicators of clinical care quality are pressure injuries and falls. Falls can have significant physical impacts, ranging from minor injuries like bruises to major injuries such as fractures, sprains, and severe head trauma. To assess fall risk early and implement preventive measures, this study analyzed 2,948 medical records of patients who underwent spinal and lower limb surgeries at the Veterans General Hospital in Taichung, Taiwan. Data collected included patient demographics, vital signs, health conditions, diagnoses, and medications, as well as information on their admission type and any recorded falls, to identify factors contributing to inpatient falls and to establish early warning measures. This study accounted for patients’ history of falls during model training, followed by variable selection and outcome modeling using logistic regression and random forest methods. Results showed that logistic regression with fall history as part of the training data is an effective approach. Patients admitted by wheelchair or stretcher for spine or lower limb surgeries had an increased fall risk. Each additional year of age also increased fall risk. In patients with arthritis, the odds of falling decreased. Conversely, the use of psychotropic and antihypertensive drugs raised fall risk. While sleeping pills reduced it. Each degree increase in body temperature and poor vision were also associated with higher fall odds. These findings support improvements in patient care quality and help reduce caregiver workload by refining fall risk assessment processes.

## Introduction

In the healthcare field medical disputes or adverse events will trigger feelings of guilt or anxiety in the clinical staff at work, which will also lead to possible treatment failures by the clinical staff. Li et al. (1) considers that the use of inappropriate medications in the elderly can lead to an increased risk of falls in the elderly. Morello et al. (2) considers that patients who experience falls have significantly longer hospital stays, which will also lead to a significant increase in hospitalization findings. Chang et al. (3) concluded that patients with repeated falls were more likely to have injuries and disabilities than patients with single falls. Patients with repeated falls had a higher correlation with antipsychotic medications and other related indicators. Tsai et al. (4) suggests that in addition to multiple comorbidities, a history of falls and frailty with older patients will have a higher risk in admission.

Shin et al. (5) concluded that older women with more comorbidities during spinal surgery were prone to in-hospital falls. Steinberg et al. (6) suggest that lower limb amputees are fall-prone patients at all stages of their illness. This also makes it necessary for caregivers to target this patient for increased prevention. Poe et al. (7) published the Johns Hopkins Fall Risk Assessment Tool (JHFRAT) to assess fall risk, which relies on fixed scoring criteria. Today’s machine learning technologies can automatically recognize more complex patterns and use multiple features to optimize the accuracy of risk assessment based on large amounts of data from different patients to more effectively reduce fall risk. In this study, machine learning was used to analyze the postoperative care of spinal or lower spine surgeries to understand the important causes of patient falls during hospitalization and to provide a reference basis for clinical care. In real life, the number of fallers is far less than the number of non-fallers, which is a serious imbalance in the classification of machine learning. In past studies, the data were often balanced and then partitioned into models for training and testing, and the modeling results were very good in terms of modeling performance due to the understanding of the structure of the data before analysis. This study adds the method of adjusting the weights of categories to improve the weight loss of a few categories during the training process, so that the training process can be more sensitive to the errors of a few categories. This study also investigate whether the adjustment of different thresholds can effectively improve the model, which is also a way to make the model perform better in unbalanced data. However, when applying to new data, once there are data with a lot of difference in variables, it is easy to make a wrong judgment, and the performance result will be much lower. In terms of model comparison this study refers to the good algorithms proposed by Young et al. (8) and Huang et al. (9) for fall risk prediction and selects two of the algorithms, Logistic Regression and Random Forest, for model comparison and analysis.

The primary objective of this study was to discuss the variable of fall history, which is a record of a fall within 12 months, which may imply that the patient is potentially at risk of falling and is a specific variable, and to examine whether this variable could provide an alternative perspective on the topic of falls for a more analytical approach. The third objective is to find and model the variables that are important for falls, reducing the variables that are important for falls is an important issue that can effectively reduce the cost of data collection. The model can be done to analyze and understand the variables that are important for increasing or decreasing the risk of falls in hospitalized patients with spinal and lower extremity surgeries, these are the two main objectives of this study.

## Literature review

As the development of artificial intelligence is getting more and more advanced and artificial intelligence can be applied in various fields, it can be used to analyze the data collected in the past and develop a model that can prevent the occurrence of adverse events using algorithms, nowadays more and more applications of artificial intelligence are in the field of healthcare. Chu et al. (10) utilized the data from electronic health records and comprehensive geriatric assessment and used the algorithm of artificial intelligence to predict the risk of falls for several elderly patients. Wang et al. (11) collected data from Taiwan’s Patient Safety Reporting System for artificial intelligence model development and found that the Random Forest Model could effectively predict the severity of patient falls. Parsons et al. (12) suggested that there is a great homogeneity between data sources and predictive model development and suggested that there is a great homogeneity between data sources and predictive model development and suggested that there is an increasing number of artificial intelligence applications in the healthcare field. Great homogeneity and argued that data analysis at high dimensional data is still not sufficiently explored. Chang et al. (13) proposed a machine learning algorithm for electronic diagnosis system and argued that Random Forest can have better results. Huang (14) published a patent for the use of medical record data characterized by admission assessment forms and accident data for fall risk assessment. Narayan and Sathiyamoorthy (15) developed a heart disease prediction system using Fourier transformations, which uses three classifiers: Artificial Neural Networks, Nano-Bayesian, and Support Vector Machines, and argued that the method can provide reliable and accurate advice. Chen and Xu (16) used an artificial intelligence approach to inpatient fall prediction and concluded that the approach could reduce the workload of caregivers in assessing falls. Huang et al. (17) conducted a predictive modeling of ventilator predictive disengagement for patients on ventilators in intensive care units. They concluded that the Random Forest model can be effective for prediction. Huang et al. (18) used a deep learning AI model for predictive modeling of ventilator disengagement concluded that the model can be effectively given to clinicians for reference. Loete et al. (19) used principal component analysis to reduce the data dimensionality and concluded that Gaussian mixture models can be used for unsupervised classification of healthy or affected patients. Jahandideh et al. (20) argued that models of deep neural networks as well as random forests are quite accurate in predicting hospital falls, and that the two models share common 12 factors in terms of features that are worth considering. Jahangiri et al. (21) argued that DNN models are better than other algorithms in predicting fall risk and found that different shifts of falls are important to improve the model’s prediction. Jain and Burke (22) utilized in-ward cameras to view patients and utilized an artificial intelligence approach to send alerts and concluded that the visits may be effective in preventing falls in assisted living facilities.

## Materials and methods

### Ethical approval

This study was approved by the Institutional Review Board of Taichung Veterans General Hospital (IRB No. CE20256B). All the data were anonymized data, and informed consent was hence waived. All methods were performed in accordance with the relevant guidelines and regulations or declaration of Helsinki.

### Patient and public involvement

General ward of Taichung Veterans General Hospital, where spinal and lower limb surgeries were performed, excluded patients under 20 years of age. The dataset consists of 3,064 records, including 419 instances with a history of falls and 2,645 instances without a history of falls. This study collected a total of 33 variables, covering demographic information, health conditions, medication usage, body measurements, and vital signs. The demographic data included gender, age, height (BH), weight (BW), and body mass index recorded at the admission assessment form. Health conditions were categorized based on the ICD10 codes issued by physicians after surgery and included insomnia, poor eyesight, hearing impairment, frequent urination, diarrhea, dementia, Parkinson’s disease, osteoarthritis, pneumonia, and anemia. Medication usage, this study referenced prescription records from the nursing system, including hypnotics, psychiatric medications, diuretics, antiarrhythmics, antihypertensives, morphine, antiepileptic drugs, hypoglycemics, and antihistamines. Additionally, polypharmacy was defined as the use of two or more medications. The vital signs section included the first post-surgical measurements of systolic blood pressure, diastolic blood pressure, mean arterial pressure, heart rate, respiratory rate, and body temperature, providing a comprehensive record of physiological status.

### Research limitations

This study did not distinguish between all patients who underwent spinal or lower extremity surgery, particularly between the degenerative disease group, the fall trauma group, and the other causes of trauma group, which may have led to a gradual dilution of data on patients with fall-related injuries. Therefore, future research will focus on more finely differentiating patients with spinal or lower extremity surgeries according to the above categorization, thus providing more precise conclusions and recommendations for health care.

### Study setting, design, and ethical considerations

**Figure 1:**
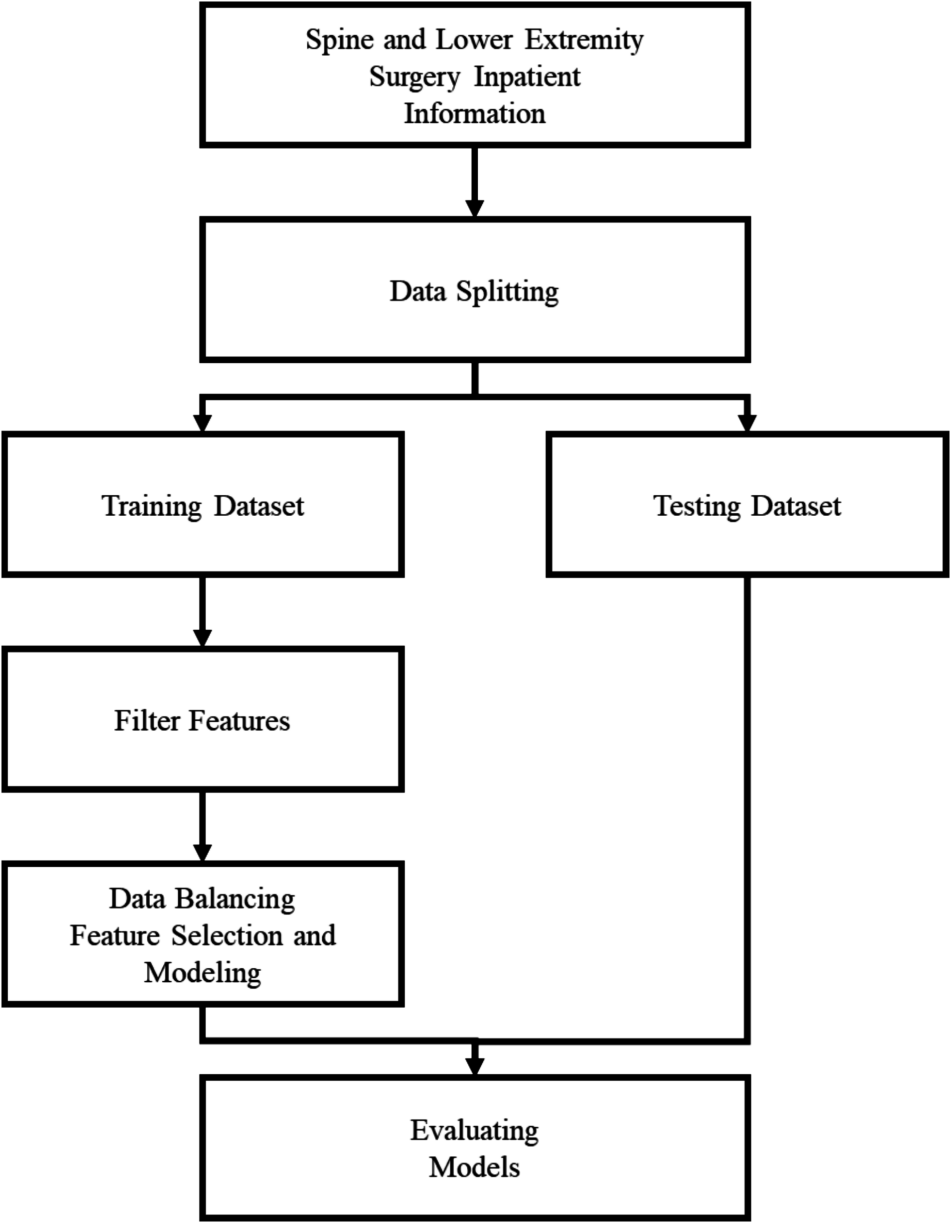
Research flow.

In this study, a total of 3,064 medical records were divided to ensure sufficient data for model training while maintaining an adequate test set for evaluation. The training dataset consisted of 2,461 non-fall cases and 409 fall cases, whereas the test dataset included 603 non-fall cases and 10 fall cases. The training set was produced by combining the variable falls and the variable falls history into a new variable, and removing the falls history variable, and the new variable was labeled as a fall risk. The Logistic regression and forward selection methods, and the importance of features in random forests, respectively, were used to screen the features. Both oversampling and undersampling are often used to balance the data. Oversampling may lead to overfitting, which affects the generalization ability of the model and reduces the accuracy of prediction, while undersampling may lead to the loss of critical clinical information, which prevents the model from adequately learning the characteristics of a few categories. To address this issue, a weighted approach was used to balance the data. By assigning higher weights to a few categories, the model can pay more attention to the categories when learning, thus improving the ability to predict falls. In this study, we set a weight of 1 for non-falls and 35 for falls to ensure that the model will not be overly biased toward the majority of categories during the training process, thus improving the prediction performance. After using two datasets and feature filtering, and after balancing the majority and minority categories, the Logistic Regression and Random Forest are built respectively, totaling four models. Step 4: The four models are evaluated using the evaluation indexes of the imbalance problem, such as Accuracy, Recall, Specificity, Precision, F1-Score, G-mean, as in Equations 1–6, and the flowchart is shown in Figure 1.


(1) $$\mathrm{Acccuracy}=\frac{TP+TN}{TP+FP+FN+TN}$$

(2) $$\mathrm{Recall}=\frac{TP}{TP+FN}$$

(3) $$\mathrm{Specificity}=\frac{TN}{FP+TN}$$

(4) $$\mathrm{Precision}=\frac{TP}{TP+FP}$$

(5) $$F1-\mathrm{Score}=\frac{2*\mathrm{Precision}*\mathrm{Recall}}{\mathrm{Precision}+\mathrm{Recall}}$$

(6) $$G-\mathrm{mean}=\sqrt{\mathrm{Recall}*\mathrm{Specificity}}$$

### Model training

**Figure 2:**
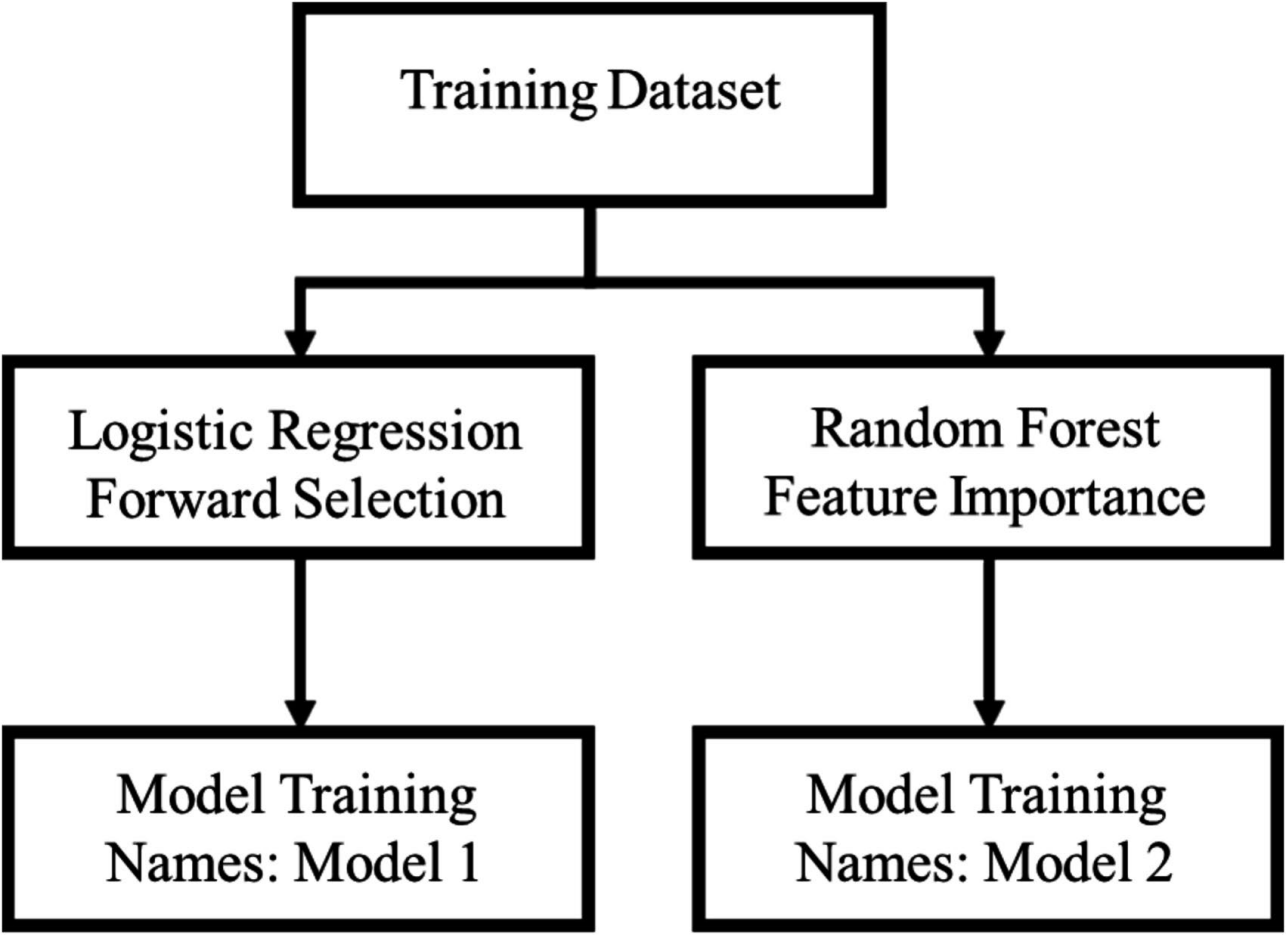
Modeling schematic.

The data processing and modeling in this study were developed using Python, and the research equipment and Python version are shown in Table 1. The parameter settings for the model training stage are shown in Table 2. After filtering the variables to build the corresponding models, Figure 2 shows the second training set, where Model3 and Model4 were generated. Considering that our problem is a highly unbalanced binary categorization problem, this study prefer to use the Recall and Specificity metrics over the Precision and F1-Score metrics in evaluating the performance of the four models. Specificity, in the discussion of this paper, represents the ratio of fallers who are judged to have fallen and the ratio of non-fallers who are judged to have not fallen, in order to achieve the purpose of better discrimination between fallers and non-fallers. In order to weigh the two indicators, the G-mean was chosen as the basis for selecting the threshold of the model on the training set, and each of them used their models and thresholds to compare the models on the test set. In order to weigh the two metrics, the G-mean was chosen as the basis for selecting the Threshold of the model in the training set, and each model and Threshold were compared with each other in the test set to compare which one has the best G-mean, and the final model was selected.

**Table 1:** Experimental equipment and software versions.

CPU	AMD Ryzen 5800 H3.20GHz
GPU	NVIDIA GeForce RTX 3060
RAM	16.0GB
Python	3.11.3

**Table 2:** Model reference settings.

Parameter name	Parameter value
Logistic regression	
C	0.1
penalty	l1
max_iter	1,000
solver	Liblinear
class_weight	Balanced
Random forest	
Bootstrap	True
class_weight	Balanced
max_depth	2
min_samples_leaf	4
min_samples_split	8
n_estimators	800

## Results

In this study, in order to understand the distribution of each variable, the variables were categorized into categorical and continuous variables. The categorical variables were presented in the form of totals and percentages, and the *p*-value was the chi-square independence test, which was used to compare whether or not there was a significant difference in the categorical variables between those who fell and those who did not fall. In the dataset, with 3,064 data, contained 419 data with fall or fall history, and 2,645 non-fall and fall history data, which was the dataset for exploring the potential fall risk, as shown in Tables 3, 4.

**Table 3:** Categorical variables—training dataset.

Category	Variables	All	Fallers	Non-fallers	*p*-value
Basic information	Male	1,308 (42.69)	156 (37.23)	1,152 (43.55)	0.017*
	Female	1,756 (57.31)	263 (62.77)	1,493 (56.45)	
Health problems	Insomnia	602 (19.65)	101 (24.11)	501 (18.94)	0.016*
	Poor eyesight	213 (6.95)	46 (10.98)	167 (6.31)	0.06
	Hearing Impaired	149 (4.86)	53 (12.65)	96 (3.63)	0.001*
	Frequent urination	336 (10.97)	114 (27.21)	222 (8.39)	0.001*
	Diarrhea	118 (3.85)	20 (4.77)	98 (3.71)	0.358
	Assistive devices	355 (11.59)	105 (25.06)	250 (9.45)	0.001*
Chronic disease	Dementia	17 (0.55)	8 (1.91)	9 (0.34)	0.001*
	Parkinsonism	17 (0.55)	7 (1.67)	10 (0.38)	0.003*
	Osteoarthritis	887 (28.95)	50 (11.93)	837 (31.64)	0.001*
Special disease	Pneumonia	27 (0.88)	9 (2.15)	18 (0.68)	0.007*
	Anemia	47 (1.53)	15 (3.58)	32 (1.21)	0.001*
Drug used	Hypnotic	513 (16.74)	81 (19.33)	432 (16.33)	0.145
	Psychiatry	477 (15.57)	117 (27.92)	360 (13.61)	0.001*
	Diuretic	244 (7.96)	75 (17.9)	169 (6.39)	0.001*
	Antiarrhythmics	311 (10.15)	67 (15.99)	244 (9.22)	0.001*
	Hypotensive	769 (25.1)	159 (37.95)	610 (23.06)	0.001*
	Morphine	2,928 (95.56)	387 (92.36)	2,541 (96.07)	0.001*
	Antiepilepsy	402 (13.12)	63 (15.04)	339 (12.82)	0.241
	Hypoglycemics	276 (9.01)	63 (15.04)	213 (8.05)	0.001*
	Antihistamine	692 (22.58)	134 (31.98)	558 (21.1)	0.001*

*: *p*-value < 0.05.

**Table 4:** Continuous variables—training dataset.

Category	All	Fallers	Non-fallers	*p*-value
Age	66 (20.0–101.0)	74.0 (20.0–99.0)	65.0 (20.0–101.0)	0.001*
BH	158.7 (114.0–190.0)	157.0 (137.0–187.0)	159.0 (114.0–190.0)	0.003*
BW	64.8 (28.2–155.0)	61.0 (30.0–155.0)	65.2 (28.2–136.0)	0.001*
BMI	25.6 (13.7–68.9)	24.1 (13.7–68.9)	25.8 (13.7–55.7)	0.001*
SBP	140 (68.0–234.0)	142.0 (75.0–226.0)	140.0 (68.0–234.0)	0.212
DBP	80 (36.0–162.0)	76.0 (36.0–157.0)	81.0 (40.0–162.0)	0.001*
MAP	100.3 (49.0–186.0)	98.0 (49.0–168.7)	100.7 (53.3–186.0)	0.005*
HR	79 (41.0–143.0)	81.0 (45.0–136.0)	78.0 (41.0–143.0)	0.001*
RR	18 (9.0–34.0)	18.0 (10.0–28.0)	18.0 (9.0–34.0)	0.622
BT	36.4 (33.9–39.2)	36.4 (34.4–39.2)	36.3 (33.9–38.8)	0.015*
Polypharmacy	2 (0.0–9.0)	2.0 (0.0–9.0)	2.0 (0.0–9.0)	0.001*

*: *p*-value < 0.05.

### Variable filtering analysis

This study discuss how to use the training set as the model training data after segmentation of the data in fall analysis, and how to find the important variables related to falls through Logistic regression and forward selection method and the importance of features in random forests.

#### Logistic regression and forward selection method

Forward Selection is a step-by-step feature selection method, which first selects the feature that has the greatest effect on the target variable, and then adds other features step by step, each time selecting the variable that can maximize the performance of the model until adding any new feature cannot significantly improve the performance of the model.

In this paper, the Logistic regression and forward selection methods are used to evaluate the selection of metrics by using the area under the ROC curve (AUC) to evaluate the model performance. Compared with the binary categorical indicators such as sensitivity, precision and F1-Score, the ROC curve can summarize the performance evaluation of binary categorical performance of the model under different thresholds without being subject to the variation of different thresholds, so the AUC is adopted as an indicator for evaluating the model performance under the use of different variables. Table 5 shows using Logistic regression and forward selection methods to evaluate the metrics with the results of the characteristic filtering of AUC. Eight variables were selected respectively, and they will be used in the Logistic modeling, respectively.

**Table 5:** Compare of feature selection from training dataset.

Number of feature	Training dataset	AUC
1	Adm kind	0.712
2	+Age	0.766
3	+Osteoarthritis	0.792
4	+Psychiatry	0.792
5	+Hypotensive	0.793
6	+BT	0.793
7	+Poor eyesight	0.793
8	+Hypnotic	0.793

#### Random forest and feature importance

In addition to modeling, the random forest algorithm also provides an assessment of the importance of each feature, which is usually based on two things: first, the average depth of the feature in all trees (the more the feature reduces the uncertainty of the model, the earlier it is used to segment the data); and second, the frequency with which the feature is used as a segmentation point in all trees (the more important the feature is, the more frequently it is used to segment the data). (the more important the feature is, the more frequently it is used to segment the data). This way of feature importance assessment provides us with a powerful tool to explain the predictions of the model and to understand the intrinsic structure of the data. This study are chosen as 11 variables selected by the model in building Random Forest, and Table 6 shows the results of the selection of the uniform variables.

**Table 6:** Results of feature importance selection.

Second training dataset	Feature importance
Adm kind	0.211
Age	0.159
Frequent urination	0.093
Osteoarthritis	0.080
DBP	0.060
Polypharmacy	0.052
Assistive devices	0.045
BMI	0.038
BW	0.033
Hypotensive	0.032
Diuretic	0.031

**Figure 3:**
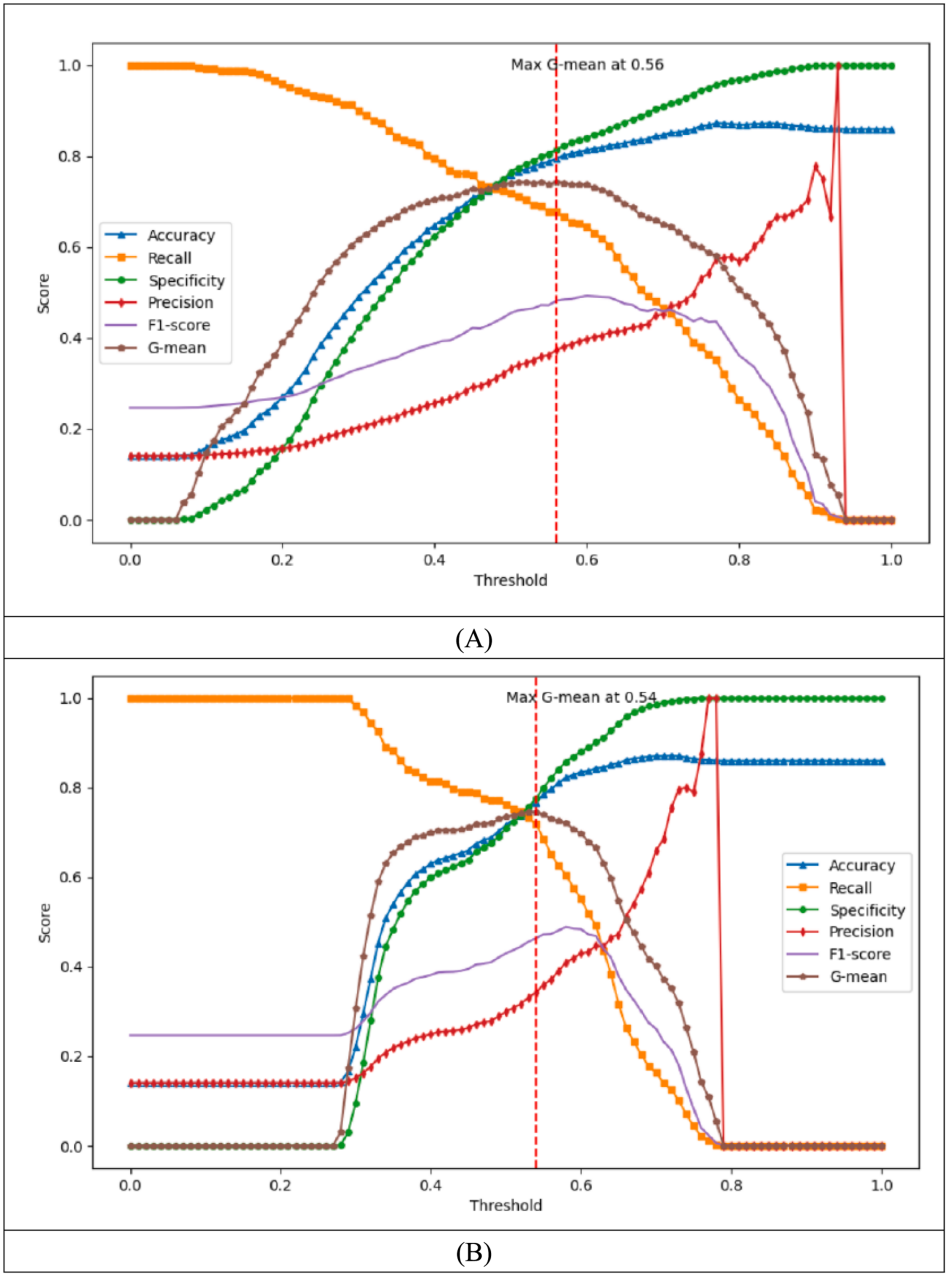
Number of two model training result.

Figure 3A presents the model built using the training set and logistic regression. This figure shows the variation in different metrics across various thresholds within the training set. When the G-mean reaches its maximum value of 0.743 at a threshold of 0.56, the corresponding confusion matrix is shown in Table 7A. At this threshold, the model’s performance metrics are as follows: Accuracy of 0.795, Recall of 0.677, Specificity of 0.814, Precision of 0.373, and F1-Score of 0.481 as shown in Table 8. Figure 3B shows the model developed using the training set and logistic regression, along with a chart displaying the changes in various metrics across different training groups within the training set. When the G-mean reaches its maximum value of 0.746 at a threshold of 0.54, the corresponding confusion matrix is provided in Table 7B. At this threshold, the model’s performance metrics are as follows: Accuracy of 0.767, Recall of 0.718, Specificity of 0.775, Precision of 0.342, and F1-Score of 0.463 as shown in Table 8. Mose et al. (23) presented a Moores Falls Scale with 78.3% in sensitivity and 83% in specificity. Compared to these traditional fall risk assessment tools, this study Model 1 exhibited superior predictive sensitivity, indicating its enhanced ability to identify individuals at higher risk of falling.

**Table 7:** Confusion matrix of models in testing groups.

(A)				(B)			
Model 3		Predict		Model 4		Predict	
		Non-falls	Falls			Non-falls	Falls
Actual	Non-falls	464	139	Actual	Non-falls	434	169
	Falls	1	9		Falls	2	8

**Table 8:** Model on test set results.

Model	Accuracy	Recall	Specificity	Precision	F1-Score	G-mean
Model 1	77.2%	90.0%	76.9%	6.1%	11.4%	83.2%
Model 2	72.1%	80.0%	72.0%	4.5%	8.6%	75.8%

After building four models earlier, with their training sets and thresholds, they were applied to the test group, Table 7 shows the confusion matrix of the four models applied to the test group, and Table 8 shows the results of their various metrics, based on G-mean as a method of selecting the models, and Model 1 was adopted as the final model.

Taken together, these observations suggest that hospitalized patients are at risk of serious injury, prolonged hospital stays, and increased healthcare costs due to falls. By combining key characteristics such as polypharmacy, osteoarthritis, diuretic use, hypotensive episodes, admission type, heart rate, diastolic blood pressure, psychiatric involvement, hypnotic medication use, age, and mean arterial pressure, the predictive model can help hospitals to adopt a proactive fall prevention strategy to reduce the potential risk. With this model, healthcare teams can shift from reactive to proactive risk management by developing precise interventions that address individual risk factors such as medication polypharmacy, hypotension, psychiatric conditions, and age, ultimately improving patient safety and reducing fall-related morbidity and mortality.

## Discussion

The use of training set represent the consideration of falls as a training set and the consideration of potential fall risk as a training set, respectively. The consideration of potential fall risk as a training set can explore whether the variables such as basic information, vital signs, health problems, chronic diseases, special diseases, and medication use of a patient with a history of falls are dramatically different from the results of the consideration of falls only. The Logistic regression and random forest models were built respectively, Model 2 with random forest can be fitted well on training, but due to the imbalance of data and the small number of fall samples, the new data is easy to predict failure once there is a big change in the situation, and the performance is worse in the test set. In the end, the second dataset with potential fall risk and the Model 1 with Logistic regression perform the best and chosen as the final models.

The Model 1 used in this study found that when patients undergoing spinal or lower extremity surgery are admitted to the hospital in a wheelchair or stretcher, the chance of falling increases compared with normal admissions. The use of admission and fall occurrence records in this study makes it easier to emphasize the significance of this variable, possibly because patients who use wheelchairs or stretchers often have other physical problems that put them at higher risk for falls. Anstey et al. (24) increased risk of falling with age is an important variable in fall prediction, and the results of the present study found that each additional year of age also increased the risk of falling. Ikutomo et al. (25) concluded that patients with arthritis are more prone to falls, and the present study found that compared to patients without arthritis, the risk of falls in patients with arthritis was slightly different. Sano et al. (26) concluded that the use of psychotropic medications leads to an increased risk of falls, and the present study found that patients undergoing spinal or lower extremity surgery who received psychotropic medications had an increased incidence of falls, compared to patients who did not take such medications. Testa et al. (27) concluded that the use of antihypertensive medication may lead to postural hypotension, syncope, or falls, and the present study found that patients on antihypertensive medication had an increased chance of falling compared to patients not on antihypertensive medication. Obayashi et al. (28) concluded that the use of appropriate sleeping pills is important in minimizing the number of falls in patients and the present study found that the ratio of falls was reduced in patients taking sleeping pills. Lord (29) concluded that poor vision leads to impaired cognition of the environment, which in turn affects the perception of gait balance, leading to an increased risk of falls, and that patients with poor vision have an increased chance of falling by a factor of one compared to patients with normal vision.

In this study, a better prediction model was obtained by using different datasets, which showed that the use of fall history and the consideration of potential fall risk contingencies is a method that can be tried in the prediction of falls, especially in the prediction of falls, which is a serious imbalance problem, so the data can be more balanced, which can increase the sensitivity of the Recall, but the precision may also be reduced as a result of this, and the consideration of different contingencies may lead to changes in data structure. When considering different variables, it may lead to changes in the structure of the data, and there are some limitations in the research of this paper. The study population only considered a sample of 2,948 medical records with spine or lower limb surgeries in Taichung Veterans General Hospital, which is not representative of all populations, and important variables such as bone mineral density and other variables that may be related to falls were not included in the study.

Although the model in this study demonstrated good efficacy in fall risk prediction, there are still some limitations. Bone mineral density (BMD) is a key indicator that reflects the strength of the patient’s bones and affects the risk of fracture after a fall. BMD is a key indicator of bone strength, which affects the risk of fracture after a fall. However, not all patients in the inpatient setting have this test performed, so BMD data were not included in this study. Future studies may consider incorporating BMD data into the model to improve the identification of high-risk patients for more accurate prevention strategies. Environmental factors are also important variables affecting the risk of falls, such as issues of non-slip flooring conditions and response time of caregivers. Existing models focus on the physiological and clinical characteristics of patients and do not fully consider the impact of the hospital environment on fall risk. Future studies could explore how to quantify and integrate these environmental factors into predictive models. To improve the generalization ability of the model.

There are very important to expand the data sources to integrate data from multiple hospitals in the future to ensure that the model maintains good predictive performance in different healthcare settings and across different patient populations. The application of multicenter data not only reduces the possible bias of single hospital data but also allows the model to learn more diverse clinical characteristics and improve applicability. Although this study provides a good prediction ability, the application of deep learning technology is worth further exploration with the development of medical big data. Compared with traditional machine learning models, deep learning can automatically extract complex data patterns and process large amounts of unstructured data. Future research should be directed toward more comprehensive data integration, not only to compensate for the lack of variables in current models, but also to improve the accuracy and clinical value of fall risk prediction through multicenter data and more advanced machine learning methods. Such advances will not only help healthcare organizations identify high-risk patients more accurately, but also enable more targeted fall prevention strategies, ultimately improving the safety and quality of care for hospitalized patients.

## Conclusion

The purpose of this study was to make a prediction of whether or not a patient hospitalized for spine and lower extremity surgery would fall. For the classification problem of binary imbalance in the field of machine learning, a research process was developed to ensure that the validation was more in line with the real situation when data analysis was performed. In the final model, for patients hospitalized for spine and lower extremity surgeries, the odds ratio of using a wheelchair or pushchair for admission to the hospital would increase the odds ratio of falls, respectively. In addition, the odds ratio for falls increased for each additional year of age. In patients with arthritis, the odds ratio for falls decreased. The odds ratios of falling were increased for patients using psychotropic drugs and antihypertensive drugs, respectively. However, the odds ratio of falling decreased for patients using sleeping pills. The odds ratio of falling increased for every degree of increase in body temperature, and the odds ratio of falling increased for patients with poor vision, and the order of increasing the risk of falling was the mode of admission, age, psychiatric medication, antihypertensive medication, body temperature, and poor vision, and decreasing the risk of falling was arthritis, and then the use of sleeping pills. These results provide a different way of thinking about the issue of falls, and the use of eight variables in the model reduces the cost of data collection compared to the original 33 variables. Through modeling, it is possible to predict in advance whether a patient is at high risk of falling and to intervene in the care of the patient, so as to achieve the effect of prevention is better than cure.

This study did not distinguish between all patients who underwent spinal or lower extremity surgery, particularly between the degenerative disease group, the fall trauma group, and the other causes of trauma group, which may have led to a gradual dilution of data on patients with fall-related injuries.

Therefore, future research will focus on more finely differentiating patients with spinal or lower extremity surgeries according to the above categorization, thus providing more precise conclusions and recommendations for health care.

## Data Availability

The original contributions presented in the study are included in the article/supplementary material, further inquiries can be directed to the corresponding authors.
